# Informational Masking in Aging and Brain-lesioned Individuals

**DOI:** 10.1007/s10162-022-00877-9

**Published:** 2022-12-05

**Authors:** Haleh Farahbod, Corianne Rogalsky, Lynsey M. Keator, Julia Cai, Sara B. Pillay, Katie Turner, Arianna LaCroix, Julius Fridriksson, Jeffrey R. Binder, John C. Middlebrooks, Gregory Hickok, Kourosh Saberi

**Affiliations:** 1 Department of Cognitive Sciences, University of California, Irvine, USA; 2 College of Health Solutions, Arizona State University, Tempe, USA; 3 Department of Communication Sciences and Disorders, University of South Carolina, Columbia, USA; 4 Department of Neurology, Medical College of Wisconsin, Milwaukee, USA; 5 College of Health Sciences, Midwestern University, Glendale, USA; 6 Department of Otolaryngology, University of California, Irvine, USA; 7 Department of Language Science, University of California, Irvine, USA

**Keywords:** Brain lesion, Masking, RMR, Stream segregation, Aphasia, Parietal

## Abstract

Auditory stream segregation and informational masking were investigated in brain-lesioned individuals, age-matched controls with no neurological disease, and young college-age students. A psychophysical paradigm known as rhythmic masking release (RMR) was used to examine the ability of participants to identify a change in the rhythmic sequence of 20-ms Gaussian noise bursts presented through headphones and filtered through generalized head-related transfer functions to produce the percept of an externalized auditory image (i.e., a 3D virtual reality sound). The target rhythm was temporally interleaved with a masker sequence comprising similar noise bursts in a manner that resulted in a uniform sequence with no information remaining about the target rhythm when the target and masker were presented from the same location (an impossible task). Spatially separating the target and masker sequences allowed participants to determine if there was a change in the target rhythm midway during its presentation. RMR thresholds were defined as the minimum spatial separation between target and masker sequences that resulted in 70.7% correct-performance level in a single-interval 2-alternative forced-choice adaptive tracking procedure. The main findings were (1) significantly higher RMR thresholds for individuals with brain lesions (especially those with damage to parietal areas) and (2) a left–right spatial asymmetry in performance for lesion (but not control) participants. These findings contribute to a better understanding of spatiotemporal relations in informational masking and the neural bases of auditory scene analysis.

**Supplementary Information:**

The online version contains supplementary material available at 10.1007/s10162-022-00877-9.

## Introduction

Several sensory and cognitive cues contribute to auditory object formation. These include a sound’s fundamental frequency, common onsets and offsets, ongoing synchronous envelopes, and spatial cues [1–6]. In multisource environments, the formation and identification of an auditory object may be degraded through masking by other auditory sources. Two basic types of auditory masking, energetic and informational, are often contrasted with each other [7–9]. Energetic masking is that in which signal and masker overlap in spectrum and time. Much of the history of research on auditory masking has focused on energetic masking, and several sophisticated signal-detection models have been developed that largely explain the mechanism underlying this type of masking [10–12]. Much less is known about informational masking, partly because it is a newer field with fewer studies and partly because of its more complex nature involving cognitive factors (e.g., selective attention and working memory) in addition to peripheral sensory mechanisms.

One useful approach to the study of informational masking is rhythmic masking release (RMR).^1^ In an RMR experiment, the observer’s task is to distinguish between two sequences of sounds that differ in their rhythmic patterns. The target sequences are presented in competition with interleaved masking sequences. These studies have shown that masking effects can be mitigated by the *addition* of sounds that are temporally synchronous with and spectrally flanking the masker sequence [13–15]. This coherence between the flanking and masking sounds leads to perceptual grouping (or fusion) of the flanking and masker sequences and better isolation of the target sequence to be detected. Spatial cues can also significantly contribute to RMR by separating a target sequence from masking sequences based on differences in their perceived locations [15–19]. The spatial RMR paradigm provides an *objective* measure of stream segregation quantified as the minimum spatial separation necessary for identification of the target sequence [18].

The study of RMR and informational masking in brain-lesioned and elderly populations is of particular interest not just for theoretical reasons but also for social and practical reasons. A common complaint among these vulnerable populations is that in relatively noisy environments where multiple individuals are simultaneously speaking (e.g., restaurants) attending to a target speaker imposes significant cognitive load that is not mitigated by simple sound amplification (i.e., by reducing energetic masking). Such adverse cognitive effects, which result primarily from informational masking, are often reported to cause mental fatigue and emotional stress [20, 21]. A better understanding of the mechanisms of informational masking in these populations can lead to improved clinical therapeutics, better hearing-aid and cochlear-implant design, reduced attentional demands in communication, and a healthier social experience.

The current study uses spatial RMR to investigate stream segregation and informational masking in three groups: people with stroke-induced brain lesions, older adults with no neurological dysfunction, and young healthy college-age students. No prior study has investigated RMR in individuals with brain lesions or in older adults. The few lesion studies that have examined the effects of spatial cues on signal detection in a multisource environment (i.e., cocktail party effect) have employed temporally and spectrally overlapping sound sources which confound measurements of informational masking with energetic masking [22, 23]. The current study uses stimuli that isolate the ability to detect informational sequences from the effects of energetic masking. The target and masking sounds comprise differing sequences of identical noise bursts that are interleaved but not temporally overlapping, facilitating measurement of informational content (i.e., temporal patterns) in the absence of energetic masking. Results showed that older and younger control groups had similar averaged RMR thresholds that were considerably lower than thresholds measured for brain-lesioned individuals. Within the latter group, those with damage to parietal regions had particular difficulty in performing the spatial RMR task. Furthermore, performance was poorer within the left-hemisphere lesioned group (nearly our entire lesion population) when the masker was presented in their left spatial hemifield, suggesting a more effective masking effect when the masker was processed by the intact right (contralateral) hemisphere.

## Methods

### Participants

Three categories of participants across four institutions participated in this experiment. The participant categories comprised 55 individuals with stroke-induced brain lesions (mean age 57.2 years, σ = 12.0, age range 29–76), 15 older adults with no neurological disease (mean age 60.1 years, σ = 7.5, age range 48–72), and 13 young college-age control participants in their late teens or early 20s. The number of participants in the older adult control group is larger than many prior auditory psychophysical studies of older adults [24–31]. Our sample size of young participants is also larger than those used in other RMR studies of young adults [15, 18].

The brain-lesioned population was recruited by and participated in the experiment at Arizona State University (*N* = 22), the University of South Carolina (*N* = 22), and the Medical College of Wisconsin (*N* = 11). The older and younger control groups were recruited by and participated in the experiment at the University of California, Irvine. All lesion participants and older adults completed audiometric tests at their respective institutions (Supplemental Table 1). In general, these participants showed some characteristic hearing loss at higher frequencies above 2 kHz. All brain-lesioned participants and age-matched older adults signed written informed consent forms approved at their respective institutions’ IRB. Participants from the younger control group were verbally consented as approved by UCI’s IRB. None of the authors served as a participant in this study.

Lesion participants were included in the present study based on the following criteria: (i) a chronic focal (6 months or more post-onset) lesion due to a stroke in either the cerebrum, cerebellum, or brain stem; (ii) no significant anatomical abnormalities other than the signature lesion of their vascular event; (iii) an absence of a history of psychological or neurological disease other than a single stroke event (e.g., no tumors, seizures, or subsequent strokes); (iv) native English speaker; (v) right-handed pre stroke; and (vi) ability to follow task instructions. All lesion participants underwent MRI scanning using a 3 T or 1.5 T MRI system at the respective testing site, and T1-MRIs and T2-MRIs with 1 mm^3^ resolution were collected and used to manually demarcate the lesion by well-trained individuals.

Locations of stroke-induced lesions were determined by visual inspection of the lesion maps generated (Fig. 4 depicts the overlap of these lesion maps after the lesion maps were transformed into Montreal Neurological Institute template space using standard procedures [32]). Inspection of the lesion maps yielded the following general categories of lesion locations: *N* = 49 left cerebral hemisphere, *N* = 1 right cerebral hemisphere, *N* = 2 bilateral cerebral hemisphere, *N* = 1 right cerebellum, *N* = 1 left cerebellum, and *N* = 1 brain stem. In the two bilateral cases, it is likely that two separate strokes led to the bilateral lesions, but the strokes occurred nearly simultaneously such that they were treated acutely during the same medical event. Thus, it is very unlikely that any between-stroke functional neural reorganization could have occurred.

Nearly all of the brain-lesion sample had neuroimaging-confirmed damage to the left cerebral hemisphere (i.e., 93%; 51 of 55; see Fig. 3). Among those 51 left cerebral-lesioned participants, the following aphasia types were identified via site-specific protocols that included the Western Aphasia Battery, Boston Diagnostic Examination, and clinical observations: Broca’s (*N* = 19), conduction (*N* = 8), anomic (*N* = 12), Wernicke’s (*N* = 1), global (*N* = 2), transcortical sensory (*N* = 2), transcortical motor (*N* = 1), and no aphasia (*N* = 6).

### Stimuli and Procedures

#### RMR Task

**Fig. 1:**
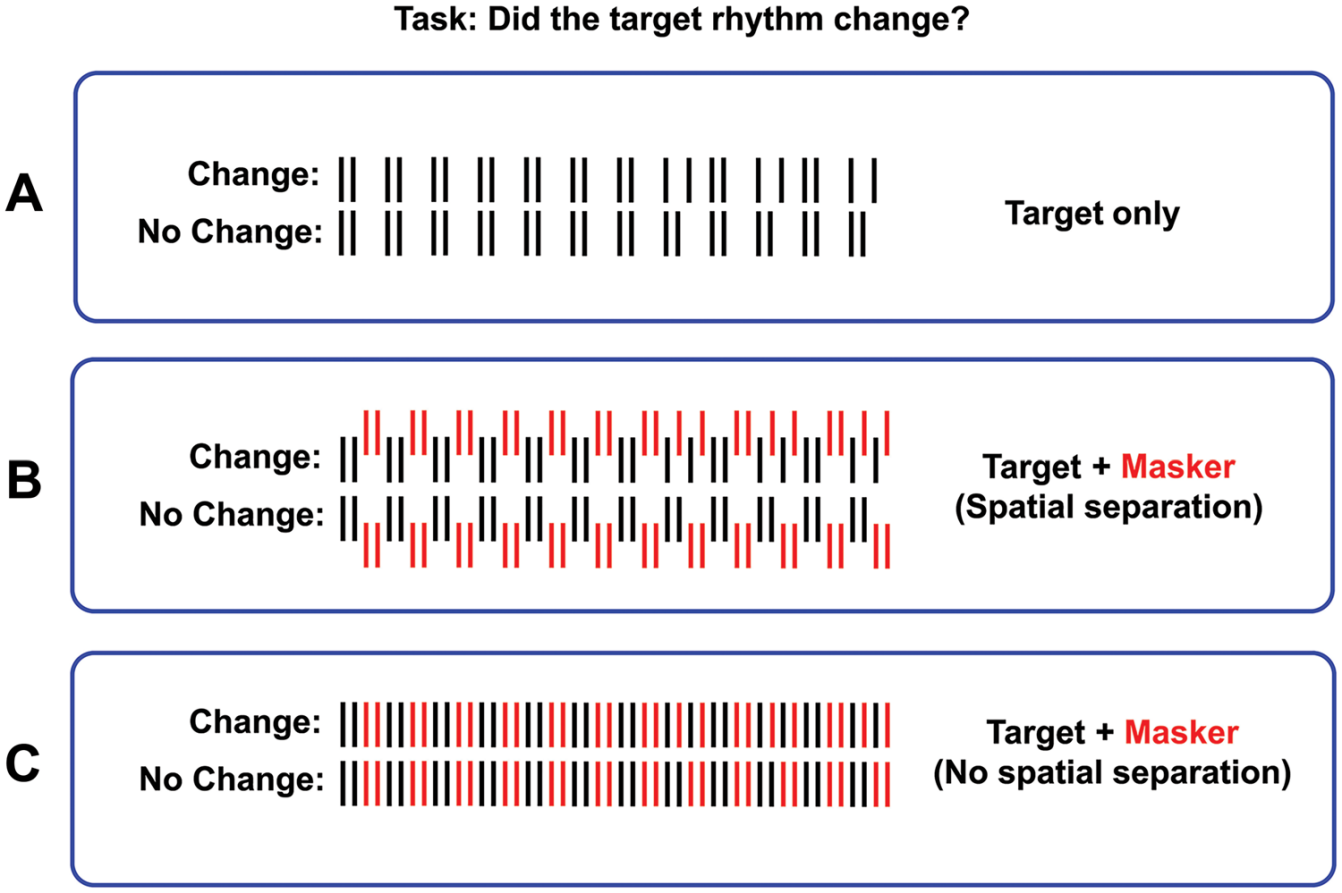
Stimuli used in the current study. On each trial either a sequence with a change or without a change was presented (**A**). The listener had to determine if the target rhythm changed midway through the sequence. The target was *always* presented in the presence of a masker sequence that precisely “filled in” the gaps in the target sequence. When there was spatial separation between masker and target sequences (**B**) the task could be performed. When there was no spatial separation (**C**), the task was impossible. RMR threshold was defined as the angular spatial separation between masker and target sequences that resulted in 70.7% correct performance

Stimuli were temporal sequences of 20-ms broadband Gaussian noise pulses generated using a Dell Latitude E5450 computer and presented binaurally through digital-to-analog converters and Sennheiser headphones (HD360 Pro) at a sampling rate of 44.1 kHz. Noise pulses were filtered through generalized head-related transfer-functions (HRTFs) to produce externalized (virtual reality) auditory percepts when presented through headphones [33]. All four research sites used identical computers, stimuli, and headphones calibrated and tested at UCI and shipped to the other 3 research sites. Figure 1 shows a diagram of target (signal) and masker sequences. The target sequence (panel A) consisted of a rhythmic pattern of noise pulses that either changed halfway through the sequence or remained the same throughout [34]. The participant’s task was to determine if there was a change in the rhythmic pattern. This single-interval design was adopted because it is a conceptually easier task for brain-lesioned participants than one that has been reported in young, non-lesion patients [18]. Panel B shows the target together with the masker sequence (red) spatially separated from each other. The target sequence was *always* presented in the presence of a masker sequence and never in isolation. The masker sequence was always complementary to the target sequence whether there was a change or no change in the target’s rhythmic pattern. This resulted in the masker pulses “filling in” the gaps in the rhythmic pattern of the target sequence. If there was no spatial separation between the target and masker sequences (panel C), the temporal sequence became uniform. This made it impossible to distinguish between the “change” and “no change” rhythmic patterns in the target sequence, resulting in chance performance. As the spatial separation of target and masker sequences increased, the task became easier. The target was always positioned at zero degrees, directly in front of the listener. The masker position was varied either toward the left or right of midline to determine the minimum spatial separation between target and masker that could be reliably detected by the participant (RMR threshold). When the masker and target sequences were combined (panel C) the aggregate pulse rates was 10 Hz (100 ms interpulse interval). The full duration of a temporal sequence that included the interleaved masker and target sequences was 4.72 s (48 pulses). For young control participants, stimuli were presented at a comfortable listening level of ~ 65 dB (A weighted) measured with a 6 cc flat-plate coupler and a sound level meter. For lesion and older adult participants, the level of the signal was adjusted individually to what was reported by the participant to be a comfortable listening level.

RMR thresholds were measured using a 2-down 1-up adaptive procedure that tracked the listener’s 70.7% correct response level [35]. Each run began with the maximum spatial separation between target and masker (90˚). Two correct responses in a row resulted in a reduction of spatial separation by a given step-size for the next trial, and an incorrect response resulted in an increase in spatial separation by the same amount. The step-sizes were progressively reduced after each track “reversal,” defined as the point at which an increase in spatial separation is followed by a decrease, or vice versa. The step-size started at 40° and then was reduced to 30, 20, and 10° where it remained for the rest of the run. Thus, near the end of a run, the spatial separation between the masker and target would change by only 10° when a change was actually required. Two thresholds were simultaneously measured by interleaving two independent threshold tracking procedures, one for the left spatial hemifield (masker location on the left of midline) and one for the right hemifield. The participant was unaware of the fact that two separate trackers were simultaneously in operation. Each interleaved track was stopped after 7 reversals. Each run took approximately 60 to 80 trials to complete and lasted approximately 10 min. Threshold was based on the averaged spatial separation at track reversal points measured at the last 6 reversals of a run. At the end of a run, two RMR threshold estimates were obtained, one for the track associated with the left spatial hemifield and one for the right. Each participant in either the younger or older control groups completed approximately 5 runs.^2^ Each brain-lesioned participant completed a single run because of secondary fatigue and task difficulty. As will be described in the “Results” section, no significant difference was observed between threshold estimates measured for brain-lesioned individuals with that measured for the “first run” of the age-matched control group. Before data collection began, all participants were given instructions about the task and performed a short pilot run on a demo program which played examples of sequences with and without a masker present, and with or without a change in the target sequence, until it was clear to the experimenter that they fully understood the task.

#### Interaural Delay Acuity Task

Lateralization thresholds [36] were also measured in a 2-interval forced-choice 2-down 1-up adaptive procedure. Stimuli were broadband Gaussian noise bursts presented binaurally through headphones with an interaural delay generated by a linear phase shift in the frequency domain across the left- and right-ear noise bursts [37, 38]. To produce a noise waveform with a linear phase shift, we first generated a Gaussian noise sample for one of the two audio channels in the frequency domain with amplitudes sampled from a Rayleigh distribution and phases from a uniform (0, 2π) distribution [39] and then linearly shifted the phase components of this burst to create the second sample. The slope of the linear phase shift corresponds to the desired interaural delay with steeper slopes associated with larger delays [40, 41]. On each trial of a 50-trial run, two successive dichotic noise bursts were presented having equal interaural delays leading to opposite ears. Each noise burst was 1 s in duration with an interstimulus interval of 500 ms. For example, a 1-s noise burst with an interaural delay of − 700 μs (leading to the left ear) was presented, followed by 0.5 s of silence, followed by a 1-s noise burst with an interaural delay of + 700 μs (leading to the right ear). This generated the percepts of two separate auditory images, one perceived on the left side of the interaural axis and the second on the right side. The order in which the two bursts were presented on a given trial was randomized. The participant’s task was to determine the order of presentation of noise bursts (left then right or right then left). Each run began with a maximum interaural delay of 700 μs (total difference of 1500 μs in the 2IFC task). This was set as the adaptive track’s ceiling value. After two consecutive correct responses, the interaural delay difference was reduced by 0.2 log units up to the 4th reversal and by 0.1 log units thereafter [42]. Following each incorrect response, the difference was increased (and the task made easier) by the same step size. Thresholds were measured as the geometric mean of the interaural delay difference at the track reversal points after the 4th or 5th reversal such that the number of remaining reversals on which threshold estimate was based would be an even number. All other conditions were similar to those used in the RMR task.

## Results

**Fig. 2:**
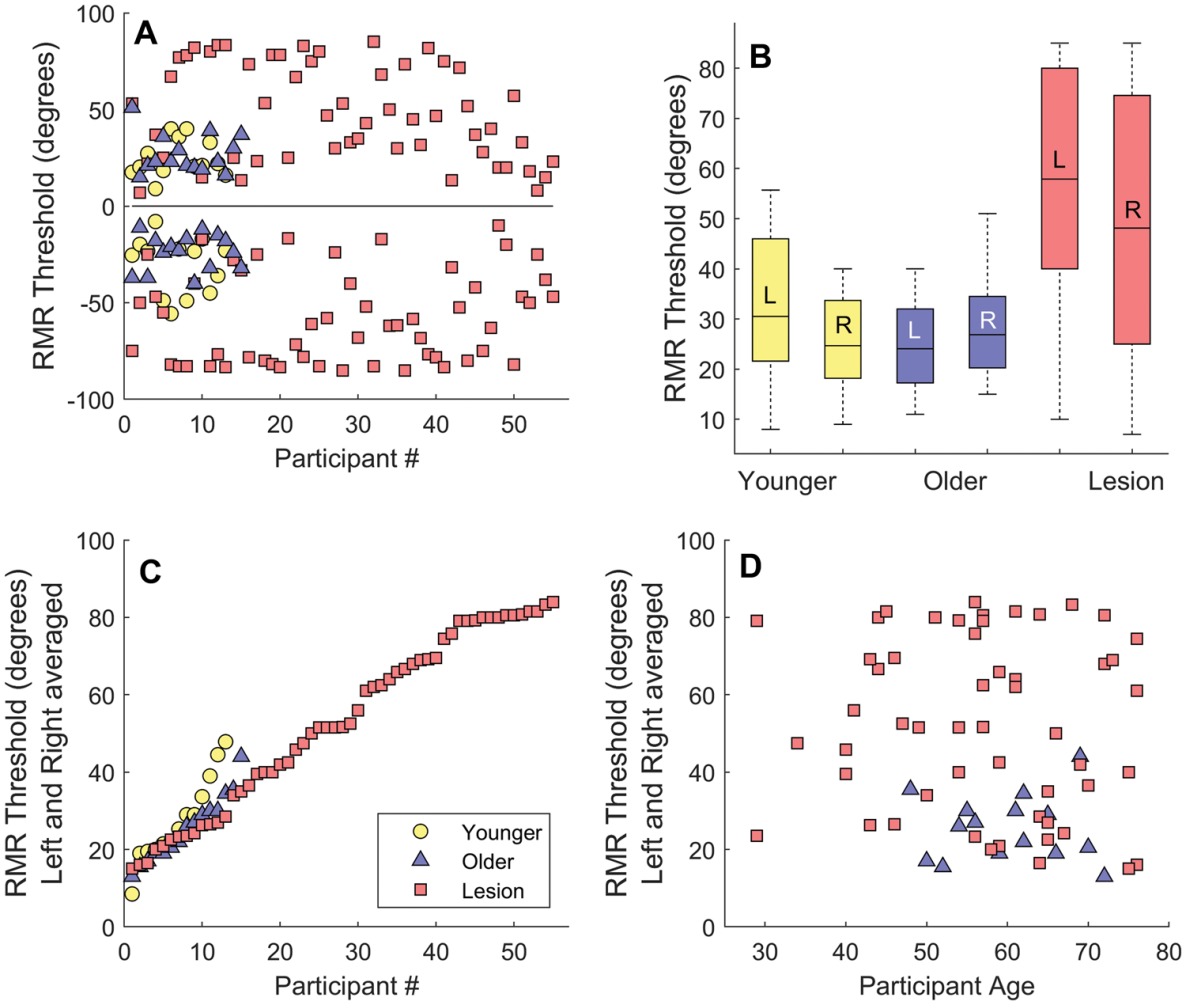
**A** RMR thresholds for the three participant groups (see **C** for legend). Thresholds are shown for the left and right hemifields (negative and positive numbers, respectively). The zero point on the ordinate designates a spatial position directly ahead of the participant. Smaller RMR thresholds near zero represent better performance. **B** Averaged RMR thresholds for the three groups (absolute values). **C** RMR thresholds averaged across the left and right hemifields and plotted in ascending order. **D** RMR thresholds for lesion participants and older controls as a function of age. No significant correlation as a function of age was observed. *N* = 55 (lesion) *N* = 15 (older adults) *N* = 13 (younger adults)

Figure 2 shows results of this experiment for the 3 participant groups. Panel A shows RMR thresholds separately for each spatial hemifield. Negative and positive values along the ordinate represent measurements for the left and right hemifields, respectively. Red squares show data from the lesion group, blue triangles from the older adult control group, and yellow circles from the young control group (see panel C for legend). Panel B shows averaged RMR thresholds from panel A for the three participant categories plotted as box plots with the central mark (small horizontal line inside each box) designating mean threshold, the edges of the box representing the 25th and 75th percentiles, and the whiskers extending to the most extreme datapoints. Kolmogorov–Smirnov tests indicated that the threshold distributions for each of the 3 groups did not statistically deviate from normality (lesion: *D*(55) = 0.115, *p* = 0.07; older control: *D*(15) = 0.124, *p* = 0.20; younger group: *D*(13) = 0.153, *p* = 0.20). Levene’s test for homogeneity of variance showed that the lesion group had a significantly larger threshold variance compared to the other participant groups (*L*(2,80) = 14.59, *p* < 0.001). Welch’s test for populations with unequal variances was therefore used to compare performance of lesion participants with the other two groups, and standard ANOVA and *t* tests were used for comparison of conditions for threshold distributions with equal variances (e.g., left–right hemifield comparisons or young vs older adult groups). Results of this analysis confirmed that there was a statistically significant effect of participant population type (*Welch’s F*(2, 35.17) = 28.73, *p* < 0.001). Post hoc analysis showed that the lesion group produced significantly higher RMR thresholds than both the younger and older control groups: (1) lesion vs. young control (Levene’s test: *L*(1,66) = 11.8, *p* = 0.001; *Welsch’s t(*1,37.87) = 34.47, *p* < 0.001) and (2) lesion vs. older adult control group (Levene’s test: *L*(1,68) = 19.4, *p* < 0.001; *Welsch’s t*(1,60.34) = 54.18, *p* < 0.001). There was no significant difference between the variances of the older and younger control groups (Levene’s test: *L*(1,26) = 0.28, *p* = 0.58) and no statistically significant difference between their mean RMR thresholds (*t*(26) = 0.57, *p* = 0.57).

**Fig. 3:**
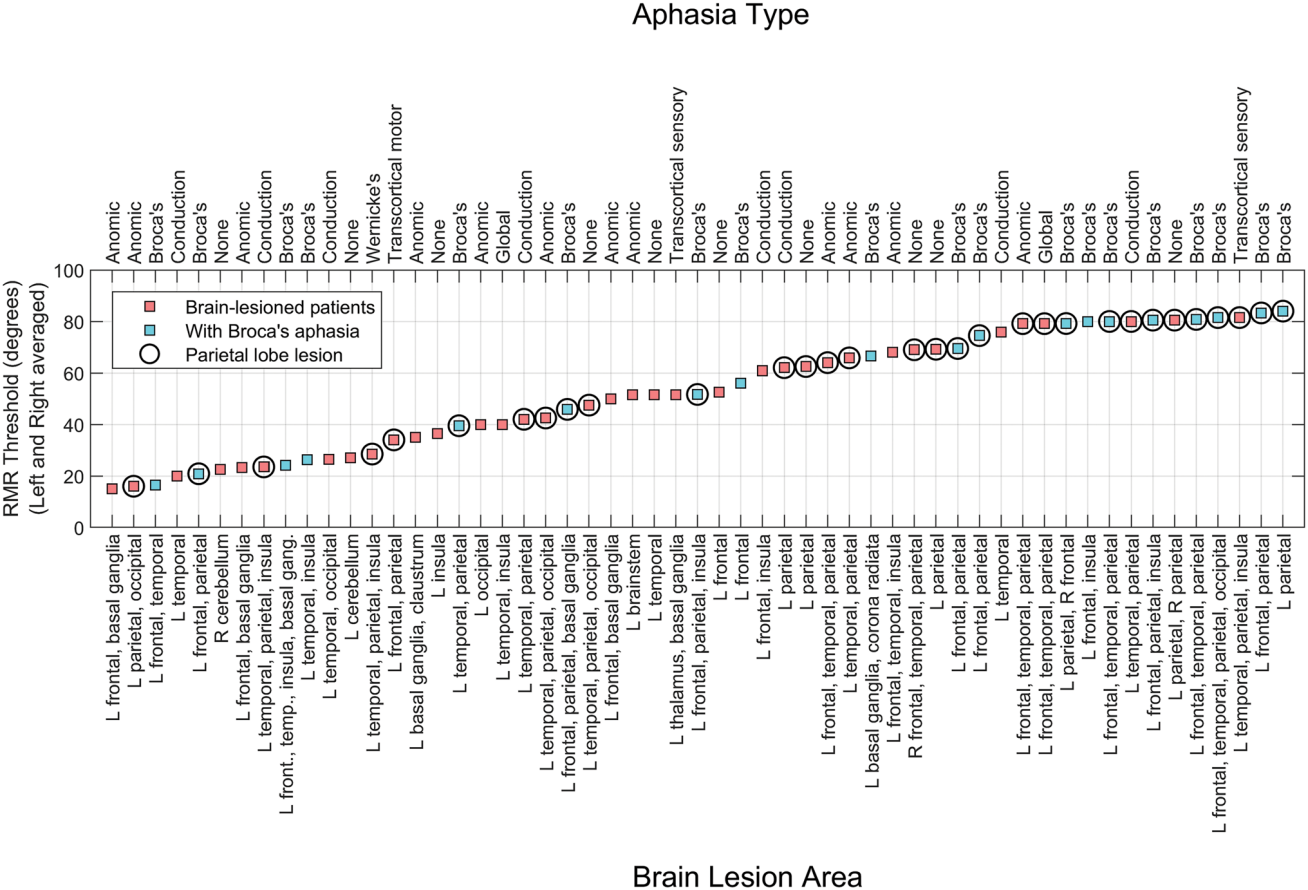
RMR thresholds for 55 brain-lesioned participants. Labels show lesion locations at the level of lobe or major structure, and aphasia description. Abbreviations: L, left hemisphere, R, right hemisphere

Nearly all brain-lesioned participants had asymmetric cortical damage to the left brain hemisphere (see Fig. 3). The lesion group showed poorer performance for maskers presented in the left hemifield (*t*(54) = 3.94 *p* < 0.001) (recall that the target was always centrally presented). The asymmetry remained statistically significant even after restricting the analysis to only those with left-hemisphere lesion (*t*(50) = 3.74, *p* < 0.001). There was no such spatial asymmetry for the younger or older control groups (*t*(12) = 1.89, *p* = 0.08, and *t*(14) = 1.17, *p* = 0.26, respectively). The left–right asymmetry in performance of the lesion group participants may be partially due to the fact that a right-sided masker, processed primarily by the damaged left (contralateral) hemisphere [43–45], is less effective in masking the centrally positioned signal (i.e., a masker presented on the left would be more effective and generate higher RMR thresholds as it is primarily processed by the intact right hemisphere). We should however caution that this asymmetry in cortical processing of sound location has been challenged by at least some studies of humans with brain lesions [46] and animal studies that have found cortical neurons with a “panoramic” view of space [47, 48]. RMR thresholds averaged across left and right spatial hemifields are plotted in ascending order in Fig. 2C. Note that some individuals in the lesion group performed as well as older and younger participants, but others had significantly higher thresholds. This is also evident in Fig. 2D where thresholds are plotted as a function of age both for the older controls and brain-lesioned participants (the younger group who were in their late teens or early 20s are excluded from this panel).

Since brain-lesioned participants completed only one run (two tracks), performance of this group was compared to the “first run” of the age-matched older adults to determine whether practice effects could explain the difference in performance (5 runs vs 1 run). Practice could not explain better thresholds for the older group compared to the lesion group as there was a highly significant difference between RMR thresholds of the older controls on their first run compared to the single-run of the brain-lesioned group (*Welch’s t*(1,29.97) = 20.04, *p* < 0.001). In addition, there was no statistically significant difference between run #1 and run #5 of the older control group (*t*(14) = 1.32, *p* = 0.208). We also considered whether hearing loss, especially in older individuals, may explain differences in performance but did not find evidence for this. Pure-tone thresholds (audiograms) are listed individually for each lesion and older adult control participant in Supplemental Table 1. Averaged pure-tone threshold for the lesion group was 16.97 dB HL, and for the older adult control group, it was 17.04 dB HL (less 0.1 dB difference), while their RMR thresholds were markedly different.^3^

**Fig. 4:**
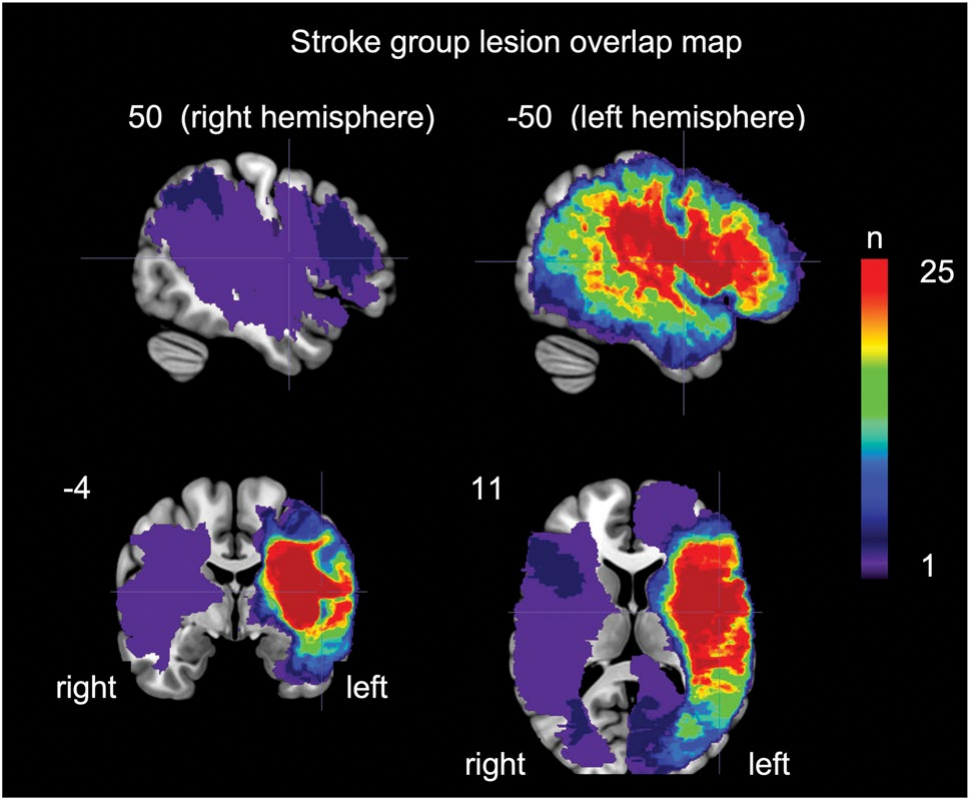
Orthogonal views of an overlap map of the areas of damage in the stroke group participants with cerebral damage, *N* = 52. Montreal Neurological Institute coordinates are provided for each slice. The area of max overlap, *N* = 25, is in the inferior frontal lobe, coordinates − 50 − 4 11. Not pictured are the single participants with left cerebellar, right cerebellar, and brain stem damage, respectively

Figure 3 shows RMR thresholds individually for all 55 brain-lesioned participants plotted in ascending order of threshold values and labeled by lesion area (lower) and aphasia type (upper). As shown in the lower abscissa, lesions were largely restricted to the left hemisphere for almost all participants except for one participant with lesion to the right-cerebral hemisphere, one with lesion to the right cerebellum and 2 with bilateral lesions as described in the “Methods” section. We should note that most of the participants were recruited based on the presence of language deficits which is primarily associated with damage to the left hemisphere of the brain.^4^ Fig. 4 shows orthogonal views of an overlap map of the areas of damage in the stroke group participants with cerebral damage.

**Fig. 5:**
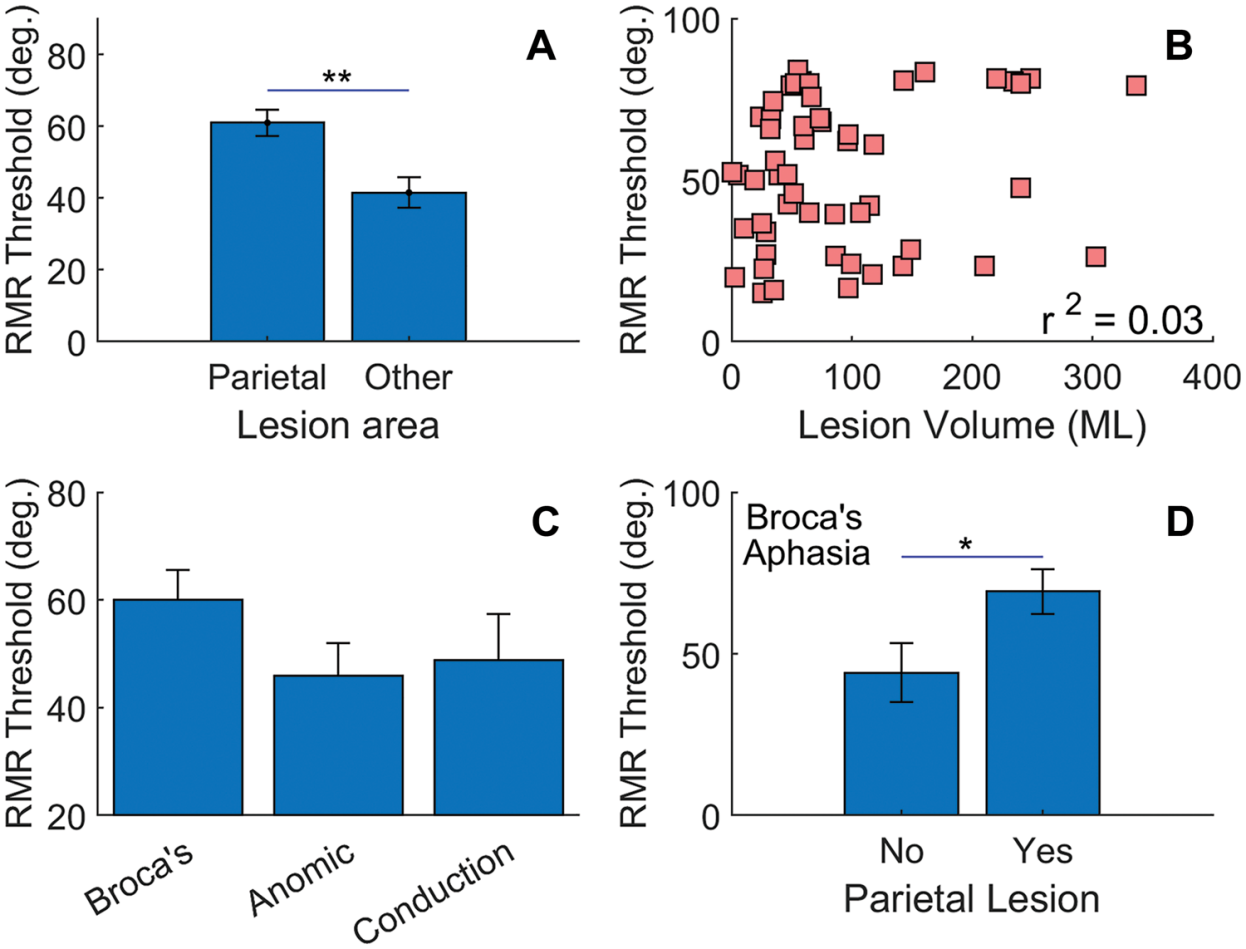
**A** RMR thresholds for those with damage to parietal regions compared to other lesion participants (*N* = 55). **B** No correlation was observed between RMR threshold and lesion volume (*N* = 54). **C** RMR thresholds for three subgroups of brain lesioned individuals (Broca’s *N* = 19, anomic *N* = 12, conduction *N* = 8. **D** Higher thresholds for Broca’s aphasics may be explained by damage to parietal regions. *N* = 19

Several human neuroimaging studies (fMRI or MEG) have implicated the parietal cortex in auditory stream segregation [49–53]. The data of Fig. 3 were further evaluated by contrasting those individuals who had lesion damage to parietal areas (circles) with participants who did not have damage to this area. Participants with a parietal lesion had a higher mean threshold compared to those with lesions to other brain regions (Fig. 5A; *t*(52) = 3.41, *p* < 0.01). Eleven of the 12 poorest performers (91%) had parietal lobe damage, while only 3 of the 12 best performers (25%) had damage to this region. Lesion volume for the parietal group was not statistically different than that for individuals with lesions to other brain regions (*t*(52) = 1.84, *p* = 0.072). Furthermore, there was no correlation between RMR threshold and lesion volume (Fig. 5B; *r*^2^ = 0.03).^5^

The three most common types of aphasia in the lesion participants were Broca’s (*N* = 19), anomic (*N* = 12), and conduction (*N* = 8). Blue symbols in Fig. 3 show those brain-lesioned individuals with Broca’s aphasia. These individuals are selectively highlighted because their RMR thresholds, as a group, appeared higher than the other two aphasic types (i.e., anomic and conduction). Eight of the 11 participants who had the highest RMR thresholds had Broca’s aphasia, though some individuals with Broca’s aphasia did produce low RMR thresholds. Figure 5C shows averaged RMR thresholds for those participants who were diagnosed with these three types of aphasia. An ANOVA on aphasia type did not reach significance (*F*(2,36) = 1.56, *p* = 0.224), nor did pairwise post hoc comparisons between aphasia types, though the difference between Broca’s and Anomic conditions approached significance: t(29) = 1.70, *p* = 0.053, one tailed. This difference, however, may be largely explained by concomitant damage to parietal regions as shown in Fig. 5D; t(17) = 2.48, *p* = 0.02.

**Fig. 6:**
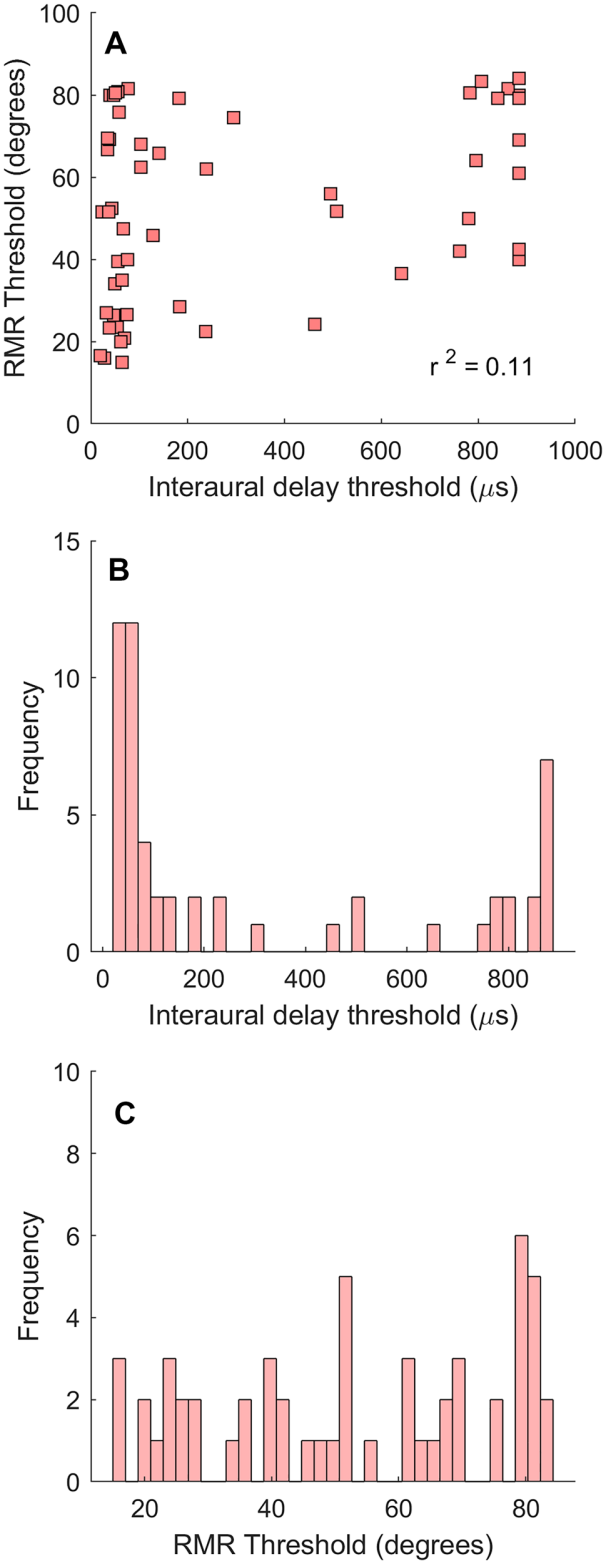
**A** RMR thresholds as a function of basic localization thresholds (interaural delays) measured from the same brain-lesioned individuals. **B** Histogram of interaural delay thresholds shows a bimodal pattern. **C** Histogram of RMR thresholds. *N* = 55

For each lesion participant, basic localization (lateralization) discrimination thresholds were also measured. Figure 6A shows RMR thresholds plotted as a function of interaural delay thresholds for each of the 55 brain-lesioned participants. There was a weak but statistically significant correlation between RMR and interaural delay thresholds (*r* = 0.33, *p* = 0.013; 11% of variance accounted for).

## Discussion

Three main aspects of the current findings are noteworthy. First, and most important, RMR thresholds were significantly poorer for the lesion group than for either control group, with an additional left–right hemifield asymmetry in performance for the lesion but not other groups. Furthermore, within the lesion group, individuals with parietal lobe damage produced the poorest performance. Second, there was a weak correlation between RMR and lateralization (spatial acuity) thresholds, with only 11% of the variance in RMR thresholds accounted for by lateralization thresholds. In fact, some participants with the lowest lateralization thresholds (under 100 μs) produced near-ceiling RMR thresholds (~ 80˚). Consistent with this finding, others have shown a dissociation between RMR and minimum audible angle (MAA) thresholds for filtered pulses [18] suggesting possibly separate but overlapping neural mechanisms underlying the two processes (informational unmasking and sound localization). They also reported that while some listeners produced significantly larger RMR thresholds compared to their MAA thresholds, other listeners produced very similar thresholds, suggesting that they were able to perform spatial stream segregation at the limits of the spatial resolution of the system. Third, the older population of control participants with no neurological dysfunction produced RMR thresholds nearly identical to those seen for the young control group. This suggests that perhaps performance on this task is largely dependent on the dominance of low-frequency interaural cues [18, 54–56] that are unaffected by the characteristic high-frequency hearing loss at old age. We suspect, however, that while we did not observe an effect of aging on RMR within the age range tested, it is likely that at some stage of advanced age, spatial stream segregation will be negatively affected similar to the adverse effects of aging in a number of other binaural tasks [57].

Auditory stream segregation involves complex cortical mechanisms at multiple structural and functional hierarchies from the auditory cortex [19, 34, 50, 58, 59] to higher centers [52, 53, 59, 60]. Both bottom-up automatic and top-down attentional processing have been implicated in stream segregation [6, 60, 61]. Human neuroimaging studies have identified the parietal cortex as particularly important to perceptually segregating auditory streams [49–53]. For example, an fMRI study [49] found that the intraparietal sulcus (IPS) is more active when two auditory streams are perceived instead of one in an ambiguous (bistable) auditory object comprising two concurrent streams.^6^ This suggests that the IPS could potentially play a role in separating two streams of sounds such as separating the target from masker sequences in the current RMR study. The streams used by Cusack were repeated triplets of interleaved low and high pitches presented at different tempos. The *same* stimulus was sometimes perceived as a galloping rhythm (i.e., a single auditory object) and sometimes as two concurrent streams (two objects). They referred to these two percepts as “horse” or “Morse” precepts, with greater IPS activity during the latter.

Another fMRI study [50] also used bistable auditory sequences to study the neural bases of stream segregation. They used two interleaved sequences (similar to our study) that were *spatially* separated via interaural level differences (ILDs) calibrated individually for each participant to behaviorally produce 50% reports of split streams and 50% reports of a unitary (or grouped) stream. Cortical activity measured using fMRI was stronger in the parietal cortex when a split stream was perceived relative to times when a unitary object was reported for the same physical stimulus. Other neuroimaging studies have also implicated the parietal cortex in auditory stream segregation [52, 53, 62]. Poorer performance in the RMR task by the parietal-lesioned individuals in our study is consistent with findings from these prior studies that have shown a critical role for parietal areas and, especially the IPS, in stream segregation, perceptual organization, and auditory “figure-ground” separation. The parietal lobe’s role in object segregation and integration is not exclusive to the auditory domain but extends to other modalities like vision and touch [63–65], as well as across modalities (audiovisual and visuotactile [66–69]).

Is the poorer performance by the lesion group related to impaired stream segregation or to an inability to process rhythms in general? The RMR task used here, by its very nature, combines these two processes. Stream segregation is a broader perceptual phenomenon that may be based not just on rhythmic patterns but also on other stimulus dimensions such as spatial separation and frequency content [70]. For example, two perceptual streams could be formed by an isochronous sequence of tones that alternate in frequency even when originating from the same location (e.g., 500, 1000, 500, 1000,… Hz). The fact that a large proportion of our lesion participants (80%) can perform the RMR task at better than chance levels (though with elevated thresholds) when the target and masker rhythmic sequences are spatially separated suggests that the impairment is not exclusively a rhythm-encoding deficit. While the rhythms *remain the same*, the task becomes easier when the spatial separation between target and masker sequences increases. This suggests that the deficit is not simply a rhythmic-encoding issue but perhaps a broader impairment of the ability to segregate perceptual streams of information. The possibility cannot, however, be excluded that there were differences in rhythm perception ability that contributed to the overall difference.

As noted above, we also found a weak correlation between RMR and lateralization thresholds. There is psychophysical evidence that localization processes may involve different brain mechanisms than those involved in RMR and stream segregation [18, 71], and prior studies with brain-lesioned individuals have shown a dissociation between abolished explicit use of auditory spatial cues in localization and preserved implicit use of spatial cues in release from masking [72]. The correlation between RMR and localization thresholds, however, while consistent with prior findings [18] should be interpreted with caution. RMR stimuli were processed through HRTFs and presented in a single-interval design, whereas interaural delay thresholds were measured for unfiltered sounds in a 2IFC design. Listeners in the RMR task had access to the full set of interaural and spectral profile cues, while they only had access to interaural delay cues in the lateralization (acuity) task. One interesting finding shown in Fig. 6B is the bimodal pattern of the threshold distributions for interaural delays. This is not observed for the distribution of RMR thresholds which appear to be more uniformly distributed (Fig. 6C). It is unclear why RMR thresholds would be more graded than the bimodally distributed localization thresholds. Perhaps this may be related to the more complex cues carried by RMR stimuli, as well as the higher-order task requirement of processing a pattern of information. The more complex cue may allow listeners to utilize different aspects of the various cues to different degrees, resulting in a more graded pattern of thresholds, whereas the discrimination of an interaural delay (a single cue) may be a simpler perceptual task resulting in an all-or-none dichotomous distribution (with thresholds for listeners who cannot do the task aggregating near ceiling values). We examined whether there were any associations between these distribution characteristics and brain-lesion patterns in our participants but did not observe any relationship.

Finally, a comparison of RMR thresholds measured for the younger group using sounds presented through headphones (i.e., generalized HRTFs) to those previously measured in the free-field shows larger averaged thresholds in the current study (25°) compared to free-field measurements (8°) where sounds are presented through loudspeakers and where subjects listen naturally through their own (individualized) HRTFs [18]. Several factors may have contributed to this difference. First, use of non-individualized transfer functions may have had some effect as suggested from an earlier study [73] which showed that optimum selection of non-individualized HRTFs based on a participant’s head width and depth produces lower RMR thresholds than those reported here (14° vs 25° on average). However, even in that study, mean RMR threshold was nearly twice as high as that reported previously [18] for free-field listening (8°). Second, the experimental procedures and protocol used in the free-field study [18] were different than ours in several respects. They required listeners to distinguish between two patterns instead of detecting a change within a pattern and used a different measurement design (method of constant stimuli with 2.5° source separation) compared to the adaptive tracking method used in the current study (10° source separation). Practice and learning effects may have also contributed to these differences across studies [73]. It is unlikely, however, that any of these factors selectively affected RMR thresholds across participant groups in the current study.

Processes that underlie RMR and stream segregation are important to communication in multisource environments where competing sources of sound can mask a target sound. The ability of humans to isolate an auditory signal in such acoustically complex environments has historically been referred to as the cocktail party effect [2, 74]. Early studies of this effect focused on how spatial separation, as well as other cues to object formation (onset differences, envelope coherence, spectral content, etc.), affect energetic masking of the target. More recent studies have investigated how the informational content of masking sequences in multisource settings adversely affect the processing of temporally non-overlapping signals. Processing of these types of temporal sequences goes beyond passive coding of informational content and may have critical predictive value for processing of impending (future) speech segments and other periodic or quasiperiodic natural sounds as demonstrated in neural and psychophysical forward entrainment [75–80]. The current study shows that brain-lesioned individuals are particularly vulnerable to interfering sequences of sounds that mask the pattern of temporal information in a target sound. Adding a linguistic component (e.g., conversation in a crowded restaurant) may make things even more challenging, especially for people with aphasia. The development of therapeutic strategies specific to encoding temporal sequences may therefore be useful to this population, especially in environments where competing sounds may interfere with the informational content of a signal.

## Supplementary Information

Below is the link to the electronic supplementary material.

Supplementary file1 (XLSX 18 KB)

## Data Availability

Raw data and analysis programs associated with the current study are available on request.

## Footnotes

1 Informational masking has been defined in a number of different ways. Here, RMR is referred to as informational (un)masking because what is interfered with is a temporal pattern of information as is typical in studies of informational masking. While the dependent measure used in the current study is spatial separation instead of decibels, what is being masked (or unmasked) is in fact a pattern of information. Also note that there was no temporal overlap between the target and masker sound pulses and, hence, no energetic masking.

2 Each participant in the older adult group completed exactly 5 runs, while some individuals in the young control group ran fewer and some more, averaging 4.85 runs across the 13 participants and ranging from 3 to 8 runs.

3 Each of the 4 research sites had their own established protocol for measuring audiogram thresholds. Supplemental Table 1 shows pure-tone thresholds for frequencies that were common to all sites, ranging from 0.5 to 4 kHz. One site recorded only pass/fail results at each frequency (up to 4 kHz) and for each ear instead of threshold values, with a pass/fail criterion of 35 dB HL. The analysis described here is based on the 32 lesion individuals and 15 older adult participants for whom numeric audiogram thresholds were available. For each participant, we calculated the overall threshold value averaged across ears and frequencies. Other than the nearly identical mean hearing threshold value (17.04 and 16.97 dB HL), the standard deviations of audiogram thresholds were also similar (9.8 and 7.8 dB for the lesion and older adult controls, respectively). Obviously a more nuanced analysis that considers the effects of loss at different regions of the spectrum may provide some additional insights. For example, it has been suggested that RMR thresholds are more severely influenced by rhythmic information in the low-frequency regions of the spectrum [16]. Examination of performance for four of our lesion participants with the poorest low-frequency hearing thresholds did not reveal any noticeable pattern, as even in these worst cases their low-frequency loss was not severe and their RMR thresholds fell within a wide range of performances. Audiograms were not measured for the young adult group who self-reported normal hearing.

4 This study is part of a larger project aimed at measuring the effects of auditory processing deficits on aphasia.

5 For one participant, L19 (see Supplemental Table 1), we have MRI confirmation of a small lesion in the brainstem. We did not, however, compute a lesion volume for this participant or map the lesion into standardized space because lesion mapping in normalized space procedures is not optimized for brainstem lesions. Furthermore, combining cerebral and brainstem lesion volumes is not considered ideal since a small brainstem lesion can cause major impairment, whereas a lesion of the same size in the cerebrum typically has little effect and may even go unnoticed.

6 A bistable “image,” often used in studies of stream segregation, is a stimulus that is sometimes perceived as one type and sometimes as another (e.g., Necker cube, or young/old woman illusion in vision). In the auditory experiments described here, the ambiguous sound is sometimes perceived as two objects (two different streams of sound, each with its own identity) or as one coherent complex object (a single stream of sound).
